# Advancement of the German version of the moral distress scale for acute care nurses—A mixed methods study

**DOI:** 10.1002/nop2.91

**Published:** 2017-09-04

**Authors:** Michael Kleinknecht‐Dolf, Elisabeth Spichiger, Marianne Müller, Sabine Bartholomeyczik, Rebecca Spirig

**Affiliations:** ^1^ Department of Nursing and Allied Health Care Professionals University Hospital Zurich Zurich Switzerland; ^2^ Faculty for Health School of Nursing Science University Witten/Herdecke Witten Germany; ^3^ Directorate of Nursing Medical‐Technical and Medical‐Therapeutic Areas, Inselspital Bern University Hospital Switzerland; ^4^ Nursing Science Faculty of Medicine Department Public Health University of Basel Basel Switzerland; ^5^ Institute of Data Analysis and Process Design School of Engineering Zurich University of Applied Sciences Winterthur Switzerland

**Keywords:** ethics, hospital, instrument development, mixed methods design, monitoring, moral distress, nurses, nursing, psychometrics, questionnaire

## Abstract

**Aim:**

Moral distress experienced by nurses in acute care hospitals can adversely impact the affected nurses, their patients and their hospitals; therefore, it is advisable for organizations to establish internal monitoring of moral distress. However, until now, no suitable questionnaire has been available for use in German‐speaking contexts. Hence, the aim of this study was to develop and psychometrically test a German‐language version of the Moral Distress Scale.

**Design:**

We chose a sequential explanatory mixed methods design, followed by a second quantitative cross‐sectional survey.

**Methods:**

An American moral distress scale was chosen, translated, culturally adapted, tested in a pilot study and subsequently used in 2011 to conduct an initial web‐based quantitative cross‐sectional survey of nurses in all inpatient units at five hospitals in Switzerland's German‐speaking region. Data were analysed descriptively and via a Rasch analysis. In 2012, four focus group interviews were conducted with 26 nurses and then evaluated using knowledge maps. The results were used to improve the questionnaire. In 2015, using the revised German‐language instrument, a second survey and Rasch analysis were conducted.

**Results:**

The descriptive results of the first survey's participants (*n *=* *2153; response rate: 44%) indicated that moral distress is a salient phenomenon in Switzerland. The data from the focus group interviews and the Rasch analysis produced information valuable for the questionnaire's further development. Alongside the data from the second survey's participants (*n *=* *1965; response rate: 40%), the Rasch analysis confirmed the elimination of previous deficiencies on its psychometrics. A Rasch‐scaled German version of the Moral Distress Scale is now available for use.

## 1 INTRODUCTION

Nurses may experience moral distress if perceived constraints prevent them from acting in accordance with their ethical judgement (Bentzen, Harsvik, & Brinchmann, 2013; McCarthy & Gastmans, 2015). The underlying motivation for the present study on moral distress among nurses was Switzerland's 2012 introduction of a diagnosis‐related group (DRG)‐based payment system (SwissDRG AG, 2010). The implementation of new reimbursement systems commonly leads to organizational restructuring, which can increase the situations where nurses experience moral distress (Rice, Rady, Hamric, Verheijde, & Pendergast, 2008; Zuzelo, 2007).

### 1.1 Background

The literature describes moral distress in nurses as a phenomenon occurring where a nurse knows what action would be correct based on his or her professional ethical principles, but for various reasons is unable either to act in accordance with these principles or to prevent potential harm (Epstein & Hamric, 2009; Fenton, 1988; Wilkinson, 1987/88). In contrast to other forms of stress, affected nurses feel that their personal moral integrity is threatened or harmed, leading them to experience moral distress (Corley, 2002; Hardingham, 2004; Webster & Baylis, 2000).

In 2010, when this study was under development, no German‐language conceptual definition existed for moral distress as experienced by nurses. For this reason, we settled on the following literature‐based working definition (translated from German):

Moral distress describes the burden felt by a nurse who believes he or she knows what the professionally ethical behaviour would be in a particular care situation but, due to impediments, is unable to act accordingly (Kleinknecht‐Dolf et al., 2014; Spirig et al., 2014).

According to this definition, the principles and values associated with moral distress are of the utmost importance. For this reason, the professional ethical principles delineated by the Swiss Association of Nurses served as the foundation for our definition (Schweizer Berufsverband der Pflegefachfrauen und Pflegefachmänner (SBK), 2013):

Professional ethical principles describe the objective to offer professional, high‐quality, safe and equitable care. Patients shall be protected from harm, their needs, preferences and resources shall be respected and they shall be supported in reaching their health‐related goals (Kleinknecht‐Dolf et al., 2014).

Professional ethical values are embedded in cultural and contextual factors (Clark, 1997; Horton, Tschudin, & Forget, 2007). It follows that this is also the case for the ethical decision‐making associated with moral distress and its impact on personal experience (Goethals, Gastmans, & Dierckx de Casterle, 2010; Varcoe, Pauly, Webster, & Storch, 2012).

Individual factors, factors relating to the work environment as well as those relating to a particular practice setting may trigger moral distress (Hamric, Davis, & Childress, 2006). Whether or to what extent a nurse experiences moral distress depends primarily on his or her moral resilience (Lützén & Ewalds‐Kvist, 2013; Monteverde, 2014; Rushton, 2016).

Depending on the effectiveness of the affected nurse's coping strategies, moral distress may lead to either psychological or physical symptoms (Hamric, Borchers, & Epstein, 2012; Huffman & Rittenmeyer, 2012; Schreuder et al., 2012). Additionally, the sense of burden can lead to job dissatisfaction or even the desire to leave the position or even the profession (Huffman & Rittenmeyer, 2012; Rushton, Kaszniak, & Halifax, 2013). Affected nurses may also withdraw emotionally from patient interactions and relationships in an effort to protect themselves (De Villers & DeVon, 2013; Evanovich Zavotsky & Chan, 2016; Whitehead, Herbertson, Hamric, Epstein, & Fisher, 2015). This may manifest itself as intolerance towards patients or the avoidance of certain interventions (Corley, 2002; Gutierrez, 2005; Hamric et al., 2006), negatively impacting the quality of treatment and care.

Given its many possible causes, the prevalence of moral distress is high. According to recent studies, nearly 47% of nurses in acute care hospitals often experience situations that trigger moral distress (Kleinknecht‐Dolf, Spichiger, et al., 2015; Ulrich, Lavandero, Woods, & Eerly, 2014; Woods, Rodgers, Towers, & La Grow, 2015).

Considering the effects of moral distress on the nurses affected by it, their patients and their organizations, the literature recommends internal monitoring of situations that commonly trigger moral distress (American Association of Critical‐Care Nurses (AACN), 2008; Pendry, 2007; Wilson, Goettemoeller, Bevan, & McCord, 2013). At the time this study was developed, no German‐language instrument had been published for institutional measurement of moral distress amongst nurses in acute care hospitals.

Hence, this study's aim was to develop an easily understandable, valid instrument for measuring and monitoring moral distress amongst nurses on inpatient units in Swiss acute care hospitals.

## 2 THE STUDY

### 2.1 Methodology

#### 2.1.1 Design

A mixed methods design was chosen, starting with an initial cross‐sectional survey, followed by a qualitative, explanatory substudy and a second cross‐sectional survey (Creswell & Plano Clark, 2006). This type of design is well‐suited for developing a conceptual understanding both of particular phenomena and of the instruments used to measure them (Creswell, Plano Clark, Gutmann, & Hanson, 2003; Onwuegbuzie & Collins, 2007). To adapt the English‐language moral distress scale to our needs, we decided on this sequential explanatory design (Creswell & Plano Clark, 2006; Ivankova, Creswell, & Stick, 2006).

**Figure 1:**
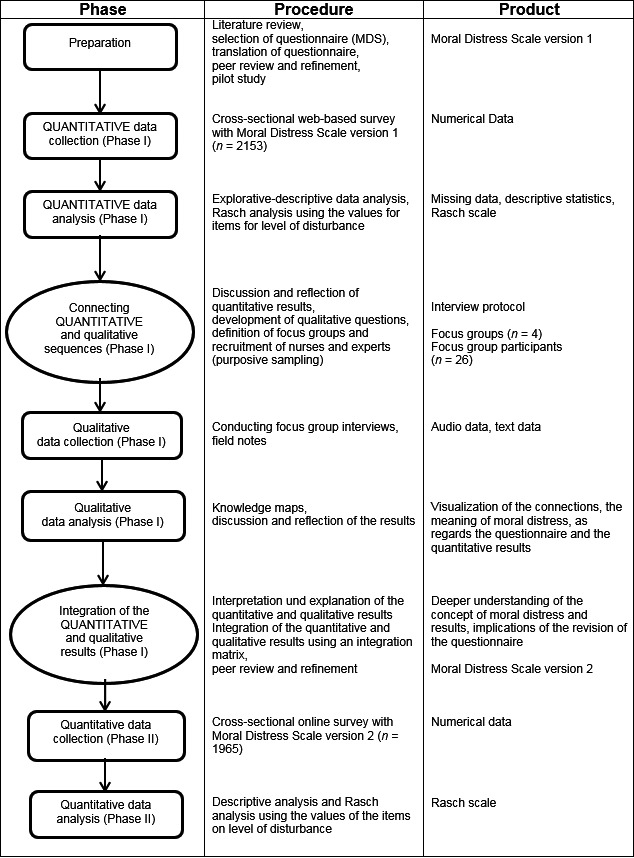
Flow chart of the sequential explanatory design procedures with repetition of the quantitative cross‐sectional survey in accordance with Ivankova et al. (2006)

During the study's initial development phase, an established moral distress scale for nurses was identified in the literature, translated and tested via a pilot study (preparation). The quantitative phase that followed (quantitative phase I) consisted of a web‐based cross‐sectional survey carried out using questionnaire version 1. Based on the results, qualitative study phase focus group interviews were carried out (qualitative phase I). The quantitative and qualitative results were then systematically integrated and interpreted (Creswell & Plano Clark, 2006; Zhang & Creswell, 2013). The information gained was used to refine the German‐language version of the questionnaire to its version 2 (Creswell, Klassen, Plano Clark, & Clegg Smith, 2011; Greene, Caracelli, & Graham, 1989), which was used for a second web‐based quantitative survey (quantitative phase II). Figure 1 shows the study's sequences.

#### 2.1.2 Methodological considerations of questionnaire development

Carried out in five hospitals in Switzerland's German‐speaking region, our research was part of a larger study aimed at developing a tool to monitor nursing‐relevant context factors in hospital work environments. One of the monitoring model's underlying context factors is moral distress (Spirig et al., 2014). In our planning phase, we identified an established American instrument for measuring this factor in nurses in acute care hospitals. Consequently, while developing the questionnaire, our focus was on producing an accurate translation, adapting it culturally, modifying its content as necessary and finally, testing the German‐language version's psychometric properties. Because moral distress is a latent variable and we intended to produce an interval scale, we carried out a Rasch analysis as an alternative to the processes suggested by classical test theory (van Alphen, Halfens, Hasman, & Imbos, 1994; DeVellis, 2012). Rasch analysis belongs to the family of item response theory models and is used in constructing interval‐scaled measures of latent traits (Hagquist, Bruce, & Gustavsson, 2009). To determine face validity, the translated and modified questionnaire was submitted several times to an expert panel. Construct validity was assessed by analysing participant results (Bannigan & Watson, 2009; DeVon et al., 2007).

### 2.2 Preparation

The objective of this preparation phase was to examine, if there already exists a well examined and established instrument for assessing moral distress in nurses in acute care hospitals, which we could use as a template for our instrument.

#### 2.2.1 Choosing a questionnaire

Following an extensive literature search in autumn 2010 on the concept of moral distress in nurses at acute care hospitals and the associated instruments, Hamric's version of Corley's “Moral Distress Scale” (MDS) was chosen (Corley, 1995; Hamric & Blackhall, 2007). Of the scales identified, the MDS conformed most closely to our working definition of moral distress. It was also the one most studied and widely used by nurses in acute care hospitals. Measured by Cronbach's Alpha, its internal consistency was 0.83 (Hamric & Blackhall, 2007).

#### 2.2.2 Translation and adaptation of the questionnaire (version 1)

After obtaining the authors' consent for use of the MDS, an expert panel of three clinical nurse specialists reduced the number of questionnaire items—which was originally designed for use in intensive care—from 21 to nine, adopting only the questions relevant to all medical specialties. The remaining nine items were then translated into German using standard methods for research translations (Jones, Lee, Phillips, Zhang, & Jaceldo, 2001; Martin, Vincenzi, & Spirig, 2007).

We then supplemented the translated questionnaire with one additional item pertaining to professional ethical behaviour. The rationale behind this addition was that work‐related moral distress in nursing is indispensable conceptually linked to the relevance of nurses' professional ethical values (Bentzen et al., 2013; Corley, 2002). Each of the 10 items was then assessed by 10 clinical nurse specialists for importance, comprehensibility and feasibility.

#### 2.2.3 Questionnaire design

To aid participants' understanding, in addition to its questions, our MDS included brief definitions of professional ethical principles and moral distress.

For the item on the importance of professional ethical principles in daily business, the frequency had to be indicated on a 5‐point verbal rating scale (0 = never ‐ 4 = very often).

Similarly, each of the nine items on moral distress used the same verbal rating scale response format to assess frequency. In addition, for each of the nine items on moral distress, participants assessed their levels of disturbance on a second 5‐point verbal rating scale (0 = none to 4 = very high). For items describing situations the participants had never experienced, they were asked to indicate hypothetical levels of disturbance (Frequency = 0). In accordance with Hamric and Blackhall's guideline, it was specified also that the reporting period for each item covered the previous 12 months (Hamric & Blackhall, 2007).

#### 2.2.4 Pilot study

In April 2011, a pilot study involving 294 nurses was conducted in eight units of one of the participating hospitals. The aim was to assess the comprehensibility and apparent content validity of the questionnaire. The details of the procedure and the results have been described in a previous publication (Kleinknecht‐Dolf et al., 2014).

### 2.3 Quantitative phase I

#### 2.3.1 Objective

The objectives of this sequence were to collect data about the relevance of the professional ethical principles as well as the frequency of occurrence and the related burden of moral distress in nursing practice. In addition to measuring moral distress amongst nurses in acute care hospitals, the goal of the first cross‐sectional survey was to test the psychometric properties of version 1 of our MDS.

#### 2.3.2 Participants and procedure

In November 2011, all RNs and clinical nurse specialists (*n *=* *4950) involved in direct patient care in inpatient units (*n *=* *204) at three university hospitals and two cantonal hospitals were invited to fill out the questionnaire. The web‐based cross‐sectional survey was conducted according to current European guidelines for “Good Clinical Practice” (European Medicines Agency, 2002). Details of the procedure are described in an earlier publication (Kleinknecht‐Dolf, Spichiger, et al., 2015).

#### 2.3.3 Data analysis

A descriptive data analysis was carried out in spring 2012 using SPSS, Version 18 (SPSS INC, 2009). For psychometric testing, the items including disturbance assessments were subjected to a Rasch analysis using RUMM2030 (Andrich, Lyne, Sheridan, & Luo, 2010). For this analysis, we used only responses of nurses who had actually experienced the given moral distress‐inducing situations (frequency >“never”).

### 2.4 Qualitative phase I

#### 2.4.1 Objective

The objective of this phase was to gather more insights about the constituent elements of the concept of moral distress in the given context of nursing practice as well as more elaborate information about the interpretation of the quantitative results of quantitative phase I to deepen our understanding of the concept.

#### 2.4.2 Participants and procedure

Drawing on the results of the quantitative data analysis, four focus group interviews were carried out in the autumn of 2012. The focus groups' 26 members included RNs, clinical nurse specialists and unit managers from one of the study's participating university hospitals. To be included, prospective participants had to have participated in the quantitative survey. Participants were recruited via an invitation circulated internally in such a way that all specialty fields were represented. This type of purposive sampling is described in the literature in connection with studies using mixed methods designs for the development of concepts or instruments (Greene et al., 1989; Teddlie & Yu, 2007). The procedure for conducting our focus group interviews is described in an earlier publication (Kleinknecht‐Dolf, Haubner, Wild, & Spirig, 2015).

#### 2.4.3 Method

Each focus group interview was moderated by two researchers, following an interview guideline based on the quantitative results of quantitative phase I. In addition to discussing the importance of professional ethical principles in clinical practice, focus group participants were asked to consider the roots of moral distress. We hoped to learn, for example, whether the questionnaire fully and comprehensively described all of the most important situations that could trigger moral distress. Regarding the quantitative results, one target outcome was the groups' explanation for instances where event frequencies for an item were equal but participants indicated widely different levels of disturbance. The focus group interviews were audio recorded and field notes taken.

#### 2.4.4 Data analysis

During the focus group interviews, in addition to the moderators, a third researcher was present to analyse the participants' statements on an ongoing basis and to depict them as knowledge maps. In the focus group interview context, analytical knowledge mapping delivers a map that highlights essential terms or topics and its relationship between them as they arise (Ebener et al., 2006; Pelz, Schmitt, & Meis, 2004). The focus group participants assessed the knowledge maps at the end of each interview for completeness and accuracy. Via qualitative content analysis, each knowledge map was reduced on its core categories (Mayring, 2008). All main points of each knowledge map were compared and generalized. The generalizations were then reduced further to yield core categories. The field notes were used to better understand the points in the context in which they arose.

### 2.5 Integration of the quantitative and qualitative results of phase I

#### 2.5.1 Objective

The objective of this study sequence was to systematically integrate the results of the quantitative and qualitative phase I to strengthen our knowledge of the concept of moral distress as well as to obtain information for the further development of the questionnaire.

Immediately following the analysis of the qualitative data in summer 2013, the integration of the quantitative and qualitative results (with respect to the field notes) began. To guide the process of integration, additional research questions were formulated (Farmer, Robinson, Elliott, & Eyles, 2006). To answer these, the qualitative results were compared with the quantitative results of the individual questionnaire items by tabulating them on an integration matrix (O'Cathain, Murphy, & Nicholl, 2010). Finally, three nurse scientists familiar with the concepts of professional ethical behaviour and moral distress explored what information had been gained that might deepen the conceptual understanding of moral distress and support the questionnaire's further development.

### 2.6 Development of version 2 of the questionnaire

Based on the insights gained through the integration process, the questionnaire was proofed for content and all items examined semantically. The questionnaire was revised beginning in winter 2013. In summer 2014, version 2 of our MDS was submitted for critical review to the same expert panel that had examined version 1.

### 2.7 Quantitative phase II

#### 2.7.1 Objective

The objectives of this phase were to repeat the cross‐sectional survey of phase I. In addition to measuring moral distress among nurses in the same acute care hospitals 4 years later and to test the psychometric properties of version 2 of our MDS.

#### 2.7.2 Participants and procedure

In November 2015, for the second cross‐sectional survey, version 2 of our MDS was presented to all RNs and those clinical nurse specialists involved in direct patient care (*n *=* *4867) in all inpatient units (*n *=* *189) of the three university hospitals and the two cantonal hospitals. As with the data collection in quantitative phase I, the questionnaire was administered in electronic form and a web‐based cross‐sectional survey conducted following the most recent European *Good Clinical Practice* guidelines (European Medicines Agency, 2002).

#### 2.7.3 Data analysis

Descriptive data analyses were carried out using SPSS, Version 22 (IBM Corporation, 2013). Again the items relating to disturbance underwent a Rasch analysis using RUMM2030 (Andrich et al., 2010). This process incorporated all responses of the participating nurses who had actually experienced the listed moral distress‐inducing situations (frequency >„never”).

### 2.8 Ethical considerations

Both in 2011 and in 2012, our proposed data collection was approved by all relevant ethics committees (KEK‐ZH‐NR: 2011‐0091). A waiver was obtained from these same ethics committees for the cross‐sectional survey in 2015 (KEK‐ZH‐NR: 82/14).

The participants of both the quantitative and qualitative phases of the study were assured that the entire participation process was voluntary and anonymous, precluding any inference or identification of any individual participant from the results. Participants in the online survey signified their consent by clicking on an option indicating approval. Participants in the focus group interviews signed informed consent forms.

## 3 RESULTS

### 3.1 Quantitative phase I

The final survey received responses from 2153 nurses (response rate 44%). The participants' sociodemographic data are shown in Table 1. The descriptive quantitative results are shown in Table 2. These results are described in detail in an earlier publication (Kleinknecht‐Dolf, Spichiger, et al., 2015). The Rasch analysis indicated differential item functioning (DIF) of several items. This means that some subgroups of nurses responded in a different manner to these items despite equally severe levels of moral distress. The analysis also showed that participants could not differentiate sufficiently between the response options of disturbance, which resulted in disordered thresholds—the failure of respondents to use the response options in a way consistent with the level of distress being measured. In addition, the targeting was not optimal. There was a lack of very difficult items, that is, situations that are assessed as not being so distressing even by highly morally stressed persons. The results of the tests on unidimensionality and on local independence of items were satisfying, as well as it was the Person Separation Index (PSI), an index frequently used in Rasch analysis, which is similar to Cronbach's alpha.

**Table 1:** Sociodemographic data of the participants of quantitative phase I and quantitative phase II

	Participants quantitative phase I (*n* (%))	Participants quantitative phase II (*n* (%))
Number of participants	2153 (100.0%)	1965 (100.0%)
Gender		
Female	1878 (87.2%)	1722 (87.6%)
Male	221 (10.3%)	215 (10.9%)
Missing data	54 (2.5%)	28 (1.4%)
Age category		
Up to 20.0 years of age	1 (0.1%)	0 (0.0%)
20.1–30.0 years of age	674 (31.3%)	619 (31.5%)
30.1–40.0 years of age	633 (29.4%)	561 (28.5%)
40.1–50.0 years of age	513 (23.8%)	401 (20.4%)
50.1–60.0 years of age	295 (13.7%)	342 (17.4%)
Over 60.0 years of age	18 (0.8%)	33 (1.7%)
Missing data	19 (0.9%)	9 (0.5%)
Percentage of full time employment		
10%/20%	32 (1.4%)	19 (1.0%)
30%–40%	214 (10.0%)	159 (8.1%)
50%–60%	277 (12.9%)	257 (13.0%)
70%–80%	501 (23.3%)	435 (22.2%)
90%–100%	1107 (51.4%)	1083 (55.1%)
Missing data	22 (1.0%)	12 (0.6%)
Years of employment		
Up to 2.0 years	525 (24.4%)	467 (23.8%)
2.1–5.0 years	554 (25.7%)	512 (26.1%)
5.1–10.0 years	402 (18.7%)	388 (19.7%)
10.1–20.0 years	422 (19.6%)	356 (18.1%)
20.1–30.0 years	171 (7.9%)	167 (8.5%)
30.1–40.0 years	43 (2.0%)	57 (2.9%)
Über 40.0 years	9 (0.4%)	3 (0.1%)
Missing data	27 (1.3%)	15 (0.8%)
Degree		
Registered Nurse/Midwifery	1951 (90.6%)	1578 (80.3%)
BScN	123 (5.7%)	340 (17.3%)
MScN	40 (1.9%)	40 (2.0%)
PhD	2 (0.1%)	0 (0.0%)
Miscellaneous	26 (1.2%)	5 (0.3%)
Missing data	11 (0.5%)	2 (0.1%)

**Table 2:** Frequency and level of disturbance of the items of the Moral Distress Scale version 1^a^

	Frequency								Level of disturbance							
	*n* b	Proportion of answers over scale (*n*, %)					Mean	SD^c^	*n* b	Proportion of answers over scale (*n*, %)					Mean	SD^c^
		0 (= never)	1	2	3	4 (= very often)				0 (= none)	1	2	3	4 (= very high)		
Professional ethical principles																
I consciously rely on professional ethical principles when making decisions regarding patient care.	2109	10 (0.5%)	54 (2.6%)	225 (10.7%)	974 (46.2%)	846 (40.1%)	3.23	.78	**‐**	**‐**	**‐**	**‐**	**‐**	**‐**	**‐**	**‐**
Moral distress																
Provide less than optimal care due to pressure from administrators or insurers to reduce costs.	2143	170 (7.9%)	598 (27.9%)	685 (32.0%)	552 (25.8%)	138 (6.4%)	1.95	1.05	2115	115 (5.4%)	269 (12.7%)	551 (26.1%)	782 (37.0%)	398 (18.8%)	2.51	1.10
Witness healthcare providers giving “false hope” to a patient or family.	2130	425 (20.0%)	776 (36.4%)	547 (25.7%)	308 (14.5%)	74 (3.5%)	1.45	1.07	2100	283 (13.5%)	406 (19.3%)	525 (25.0%)	629 (30.0%)	257 (12.2%)	2.08	1.23
Carry out the physician's orders for what I consider to be unnecessary tests and treatments.	2136	98 (4.6%)	586 (27.4%)	714 (33.4%)	527 (24.7%)	211 (9.9%)	2.08	1.05	2111	146 (6.9%)	519 (24.6%)	676 (32.0%)	549 (26.0%)	221 (10.5%)	2.08	1.09
Avoid taking action when I learn that a physician or nurse colleague has made a medical error and not reported it.	2103	1134 (53.9%)	619 (29.4%)	230 (10.9%)	89 (4.2%)	31 (1.5%)	.70	.93	2087	446 (21.4%)	314 (15.0%)	414 (19.8%)	471 (22.6%)	442 (21.2%)	2.07	1.44
Be required to care for patients I don't feel qualified to care for.	2136	1094 (51.2%)	826 (38.7%)	157 (7.4%)	43 (2.0%)	16 (0.7%)	.62	.77	2113	725 (34.3%)	361 (17.1%)	269 (12.7%)	388 (18.4%)	370 (17.5%)	1.68	1.52
Work with nurses or other healthcare providers who are not as competent as patient care requires.	2122	416 (19.6%)	910 (42.9%)	480 (22.6%)	260 (12.3%)	56 (2.6%)	1.35	1.01	2095	311 (14.8%)	423 (20.2%)	451 (21.5%)	579 (27.6%)	331 (15.8%)	2.09	1.30
Ignore situations of suspected patient abuse by caregivers.	2090	1501 (71.8%)	394 (18.9%)	119 (5.7%)	47 (2.2%)	29 (1.4%)	.43	.81	2088	653 (31.3%)	221 (10.6%)	288 (13.8%)	396 (19.0%)	530 (25.4%)	1.97	1.60
Watch patient care suffer because of a lack of provider continuity.	2125	191 (9.0%)	645 (30.4%)	649 (30.5%)	447 (21.0%)	193 (9.1%)	1.91	1.11	2104	182 (8.7%)	418 (19.9%)	614 (29.2%)	621 (29.5%)	269 (12.8)	2.18	1.15
Work with levels of nurse or other care provider staffing that I consider unsafe.	2134	359 (16.8%)	583 (27.3%)	480 (22.5%)	406 (19.0%)	306 (14.3%)	1.87	1.30	2107	253 (12.0%)	327 (15.5%)	446 (21.2%)	565 (26.8%)	516 (24.5%)	2.36	1.32

a German items were translated for this publication

b Deviations from the overall total of 2153 resulted from participants' freedom to answer individual items

c SD: Standard deviation

The results of the Rasch analysis gave us valuable hints to improve the wording of the statements and the response scales.

### 3.2 Qualitative phase I

Table 3 shows the sociodemographic data of the 26 focus group participants interviewed following the quantitative data analysis. Most focus group participants described the questionnaire as generally comprehensible and agreed that the items' content was both important and semantically applicable. However, several noted that certain statements were imprecisely formulated or difficult to understand. Regarding completeness, participants mentioned that the questionnaire omitted several important moral distress‐inducing situations. Specifically, they cited non‐collegial collaboration, dependence on inadequate orders from physicians and the informal assumption of responsibility for other hospital workers' tasks.

**Table 3:** Sociodemographic data of the participants (*n* = 26) of the qualitative phase I

	(*n* (%))
Gender	
Female	23 (88.5%)
Male	3 (11.5%)
Age category	
25.0–40.0 years of age	7 (26.9%)
40.1–50.0 years of age	14 (53.8%)
50.1–65.0 years of age	5 (19.3%)
Percentage of full time employment	
60%–70%	2 (7.7%)
80%–100%	24 (92.3%)
Professional experience	
2.1–10.0 years	5 (19.2%)
10.1–30.0 years	19 (73.1%)
Over 30.0 years	2 (7.7%)
Position	
RN	14 (53.8%)
Clinical nurse specialist	6 (23.1%)
Unit manager	6 (23.1%)

Regarding the adequacy of the 5‐point response scale for frequency and level of disturbance, the participants explained that the assessment of how frequently a given situation occurs is highly dependent on a subjective evaluation of that situation's potential impacts. Hence, identical responses to different statements do not necessarily convey the same degree of frequency. Added to this, the employment status of the person making the assessment and the size of the unit also played roles. Therefore, several participants recommended making the response categories less subjective.

Similarly, the participants described their perceptions of the level of disturbance as dependent not only on their subjective assessment of the risk involved but also on the degree to which their own moral integrity was threatened or harmed. They also emphasized that their perception of disturbance could depend, for example, on the extent to which they were constrained from taking action, on their own state of health, on work pressures, their mood, or the length of their current sequence of working days. Here also, the focus group noted that identical values for disturbance did not convey identical meaning for each item. For this reason, they suggested that the disturbance scale should also include more specific assessment terms.

Regarding the relationship between the frequency with which a stress‐inducing situation occurs and the intensity of the disturbance associated with it, the participants described various viewpoints. They explained that, in cases where it is possible to cope with a particular situation, it is possible to keep the level of disturbance from increasing, even if the situation is ongoing or escalates. However, if this coping ability is not learned, the level of disturbance due to moral distress may increase. A more extensive description of the results of the focus group interviews can be found in a previously published article (Kleinknecht‐Dolf, Haubner, et al., 2015).

### 3.3 Results of integration of the quantitative and qualitative results of phase I

Regarding interpretation of the quantitative results on moral distress, the qualitative focus group data revealed that that the degree of frequency assigned to a particular item does not correlate consistently with the degree of distress engendered. For the same reason, the levels of disturbance assigned to different cases are not directly comparable. This fact complicates the interpretation of the results.

Applying an integration matrix confirmed that the statements on the questionnaire are applicable and relevant. However, additional items are required to cover non‐collegial collaboration, inadequate physicians' orders and informal assumptions of responsibility for other hospital workers' tasks. Table 4 shows an example of the integration procedure that led to these results. Regarding the response scale for frequency and level of disturbance, our integration matrix indicated that improvements to the response category descriptions would ease the response process and improve the validity of the results. The resulting integration and corresponding results are shown in Table 5.

**Table 4:** Example of integration of quantitative and qualitative results for individual questionnaire statements

Statement from the 2011 survey with version 1 of the questionnaire	Missing data (for *n* = 2153)	Quantitative results [*n* (%)]	Qualitative results	Integration	Statement from the 2015 survey with version 2 of the questionnaire
Item 7 “Ignore situations of suspected patient abuse by caregivers”	Frequency 63 (2.9%) Level of Disturbance 65 (3.0%)	Frequency Never (0): 1501 (69.7) 1: 394 (18.3) 2: 119 (5.5) 3: 47 (2.2) very often (4): 29 (1.3) Level of Disturbance None (0): 653 (30.3) 1: 221 (10.3) 2: 288 (13.4) 3: 396 (18.4) Very high (4): 530 (24.6)	The item is important and generally formulated in a comprehensible way. The tense used in the formulation makes it unclear whether it refers to present behaviour or to past actions. Several participants had never experienced the situation described. Some feel that the description is not precise enough, making different interpretations possible. For example, one response was: “But isn't what it meant by that, whether someone is or is not taken seriously? … That's the most difficult question for me” or another example: “The question is, when does neglect begin?” The assessment of frequency and level of disturbance varies from person to person. The same individual may even assess identical situations differently depending on their own personal circumstances or workplace environment.	The small amount of missing data and the information gained from the focus groups confirm that the item was generally formulated comprehensibly and is important. The tense has to be changed so that it is clear that it refers to an assessment of the respondent's own actions in the past. The qualitative results confirm the quantitative results, which show that this is a situation that rarely occurs. The description of neglect and abuse must be made more precise. It is noticeable that, for this statement, there are significantly more assessments for level of disturbance (value >0) than there are for frequency (value >0). This can be explained by the instruction to give a hypothetical assessment of disturbance for situations that have not occurred. The fluid nature of the assessments on frequency and level of disturbance, makes an accurate interpretation of the quantitative results difficult. The response scale must be formulated more precisely, the instructions revised and the assessment period shortened.	New formulation of the statement: “Have taken no action in instances where there were signs of possible verbal or physical abuse of patients or patient neglect”. (and supplemental reference to abuse and neglect) The response scales were with supplemented with revised qualifiers. The instructions were revised to remove the need for a hypothetical assessment and the assessment period was reduced to 3 months.

**Table 5:**
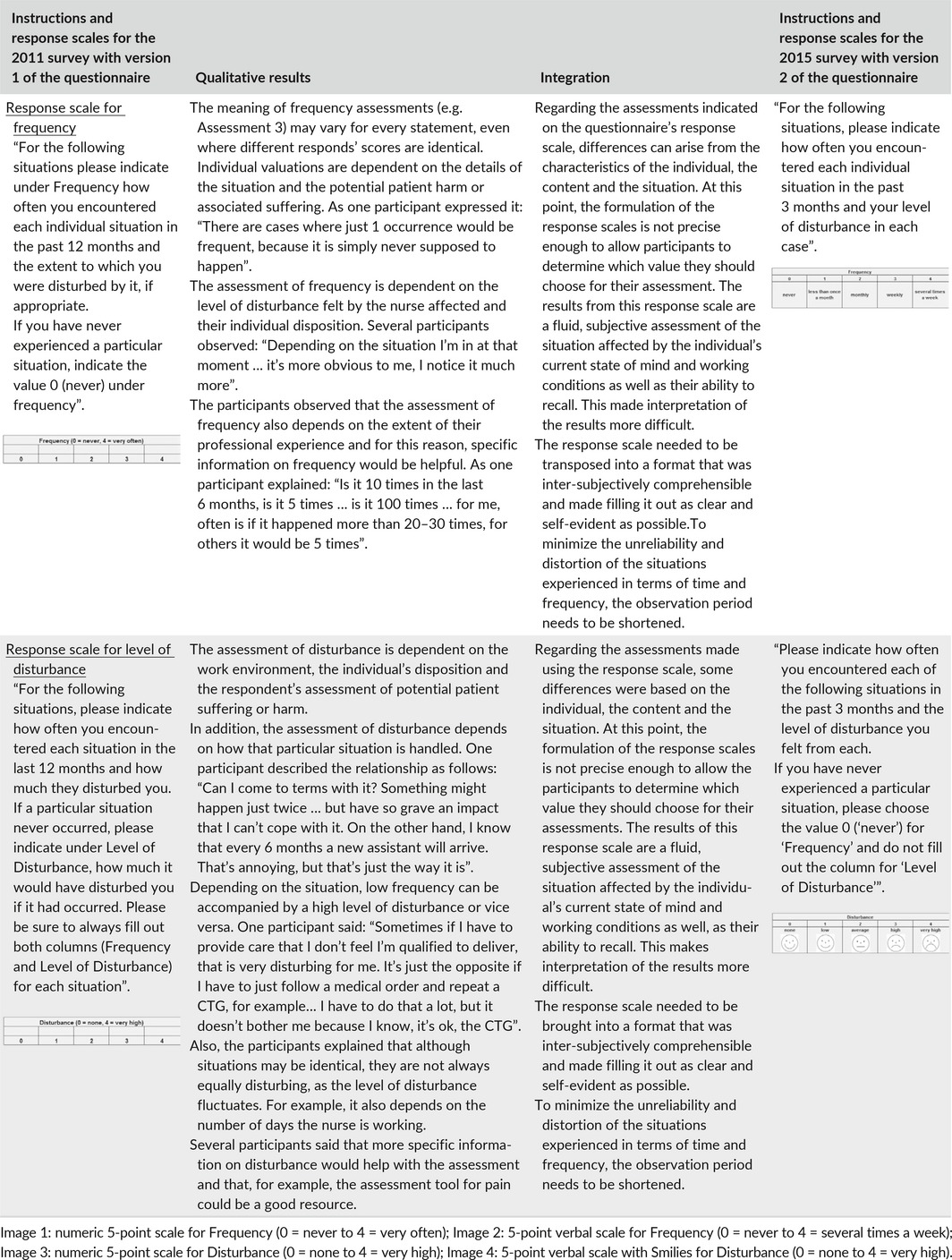
Integration of the qualitative results with instructions and response scales

### 3.4 Version 2 of our MDS

The insights gained from the integration process were used to refine all items semantically. In addition, three items (Items 10, 11 and 12) were added to the questionnaire. Finally, each response category of the 5‐point response scales for frequency and distress level was provided with new qualifiers. To the scale assessing frequency, we added clear numeric ranges for each response value. Other authors investigating moral distress have added similar qualifiers to the MDS frequency scale (De Veer, Francke, Struijs, & Willems, 2013).

We refined the wording of the scale for assessing the level of disturbance. Additionally, to each distress level response category, we added a “smiley” icon corresponding to that particular level of disturbance. While the idea to add “smileys” was proposed in our focus group interviews, other studies have also found that adults readily accept smiley icons as a graphic aid for numerical values in scales recording latent variables such as pain (Jäger, 2004; Wong & Baker, 2001). Permission to use these statistically tested icons was obtained from their author (Jäger, 2004; Jäger & Bortz, 2001).

After these adaptations were in place, version 2 of our MDS was once again assessed by a statistician and an expert panel. Based on discussions regarding these assessments, the length of the retrospective assessment was shortened from 12 to 3 months. Also, considering our new insights, we concluded, as did the original authors of the MDS (Corley, Elswick, Gorman, & Clor, 2001), that in cases where frequency was assessed at 0 (“never”), the assessment scale for disturbance should be left blank (value = missing). This technique limits assessment to disturbance arising from situations actually experienced by the respondents. The structure and revised items of version 2 of our MDS are shown in Table 6.

**Table 6:**
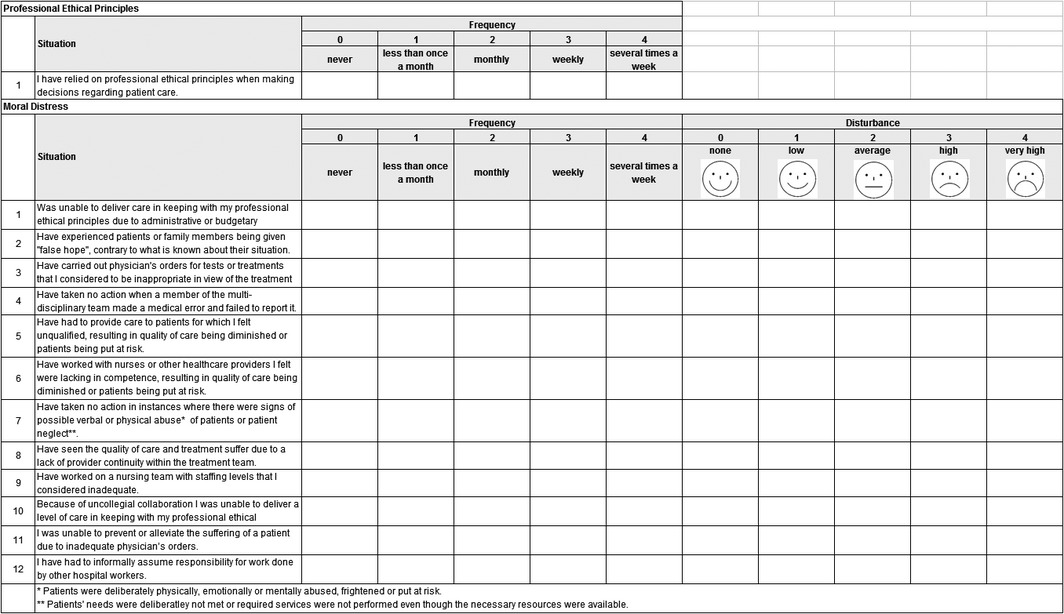
The structure and items of Moral Distress Scale version 2 on measuring moral distress in nurses at acute care hospitals

### 3.5 Quantitative phase II

In total, 1965 nurses (response rate: 40%) took part in the survey using version 2 of our questionnaire. The sociodemographic data of the participants are shown in Table 1.

The Rasch analysis of the revised items showed that all items worked (no more DIF) and that participants used the response categories in a consistent way. Once again, the results of the tests on unidimensionality and on local independence of items were good as well as it was the PSI. Targeting was also improved.

## 4 DISCUSSION

This study's aim was to develop a comprehensible and valid German‐language instrument to measure moral distress in nurses at acute care hospitals. The results of the individual phases of our mixed methods research indicate that the strategy chosen fulfilled this aim. Following translation of Hamric's MDS (Hamric & Blackhall, 2007), our addition of several items, including one on the importance of professional ethical principles, was judged adequate and appropriate by an expert panel of clinical nurse specialists, as the additions strengthen the face validity of the item set chosen (Houser, 2008).

The results of our pilot study in April 2011 showed that the translated items were fundamentally comprehensible and relevant. As an indication of the questionnaire's construct validity, the results produced by our MDS correlated with the responses expected from the participating nurses (Wampold, Davis, & Good, 1990).

A response rate of 44% (2011) and 40% (2015) for the two cross‐sectional surveys is a common response rate for this type of web‐based cross‐sectional survey (Cook, Heath, & Thompson, 2000). A response rate of at least 40% is considered a prerequisite for obtaining reliable evidence on the unit level (Kramer, Schmalenberg, Brewer, Verran, & Keller‐Unger, 2009).

Both item use and response variability are important indicators of questionnaire quality (DeVellis, 2012). Given that, for all items of our MDS, the full range of offered response options were used in both cross‐sectional surveys, with reasonable variation between respondents, the response categories represent diverse subjective assessments for the individual item statements and are adequately sensitive within the various scopes of application.

In line with similar studies, the quantitative results of our 2011 survey showed that professional ethical principles play a key role in all areas of routine nursing, with a pronounced influence on nursing practice (Bentzen et al., 2013; Kangasniemi, Pakkanen, & Korhonen, 2015). Supporting corresponding results of other studies using Hamric's MDS, the results of our nine selected items on moral distress‐inducing situations out of it show that moral distress is experienced in all practice areas, sometimes to a high degree (Fernandez‐Parsons, Rodriguez, & Goyal, 2013; Hamric et al., 2012). However, the interpretation of these quantitative results is limited by the fact that respondents also assessed levels of disturbance for situations that they did not actually experience. These hypothetical assessments distort the levels of disturbance indicated by those nurses actually affected by moral distress and complicate discussions on possible measures to prevent or reduce moral distress. For this reason, we decided to follow the former application guideline set by the original authors of the MDS and to abandon hypothetical answers of disturbance (Corley et al., 2001).

The results of the Rasch analysis of the 2011 cross‐sectional survey data provided important material with which to refine our initial translation of the MDS. In contrast to classical test theory, Rasch analysis is well‐suited to develope questionnaires involving latent constructs (van Alphen et al., 1994; Hagquist et al., 2009). The Rasch analysis indicated the need for items that even nurses with high levels of disturbance did not assess as particularly disturbing. It also indicated that the formulation of certain existing items needed revision.

Our qualitative results confirmed that the items selected from Hamric's version of the MDS were relevant and comprehensible. Moreover, the focus group participants provided three moral distress‐inducing situations not included in Hamric's MDS (Hamric & Blackhall, 2007). While other publications have described a lack of collegial collaborations and inadequate physician's orders as moral distress‐inducing situations, we know of no study that included the informal assumption of other staff members' responsibilities in this category (Huffman & Rittenmeyer, 2012; McCarthy & Gastmans, 2015). Our focus groups' inclusion of this scenario reflects their professional and cultural context. Such changes to the MDS follow the lead of studies in other work contexts that required modifications to Hamric's MDS item statements (Eizenberg, Desivilya, & Hirschfeld, 2009; Hamric et al., 2012). As described in at least one previous study on this subject (Burston & Tuckett, 2013), our focus group interviews also highlighted multiple factors influencing the experience of moral distress.

Integration of our quantitative and qualitative results identified the questionnaire content in need of revision and augmentation. Crucially, it also increased our understanding of how the frequency of particular situations relate to the levels of moral distress experienced. This relationship's effect can be positive or negative, that is, frequent exposure to a morally stressful situation can either raise or lower the level of disturbance. Previous studies have reported similarly equivocal relationships (Monteverde, 2016; Wilkinson, 1987/88; Wlodarczyk & Lazarewicz, 2011).

Variations in respondents' moral resilience or coping mechanisms may partially explain why the frequency/disturbance relationship manifests itself in opposite directions. However, our focus groups emphasized that the moral distress‐inducing situations listed are interpreted and assessed in the context of potential adverse effects on specific patients, that is, across diverse care contexts, identical frequencies can yield diverse levels of distress. For this reason, response values that are identical do not necessarily mean the same thing. Therefore, it is essential that the qualifiers provided in the response scales be as unambiguous as possible. This observation is also a strong argument for why the individual responses on frequency and disturbance should not be combined into one mathematical product which is then totalled to create an overall score intended to express the overall level of disturbance. Several studies on the MDS describe this algorithm for calculating an overall score (Hamric et al., 2012; Lazzarin, Biondi, & Di Mauro, 2012; Wiggleton et al., 2010). In contrast, our Rasch analysis showed that it is possible to generate an interval‐scaled Rasch score just from the individual responses on disturbance that represents the overall level of disturbance, making it possible to compare the total scores of individual nurses while accounting for item difficulties. The results of the frequency scale can be used to express the prevalence of each listed situation and to monitor its occurrence. Several studies have shown the use and usability of similar MDSs (Borhani, Abbaszadeh, Nakhaee, & Roshanzadeh, 2014; Kleinknecht‐Dolf et al., 2014; Piers et al., 2012).

Overall, our findings show that, through the integration of quantitative and qualitative results and in accordance with the literature, we were able to add materially to previous knowledge of the concept of moral distress, as well as to improve the structure and content of the associated questionnaire for our study context (Creswell et al., 2011). Repetition of the Rasch analysis using the data from the second cross‐sectional survey showed substantial improvements to our MDS version's psychometric properties, making it suitable for future cross‐sectional surveys of nurses in acute care hospitals.

### 4.1 Limitations

Our study has various limitations. Although the quantitative results are based on surveys at each of the five hospitals participating in the study, resource constraints dictated that the qualitative data had to be gathered from focus group interviews held at only one. The extent to which that hospital's nurses represent the views of those in the other four and the extent to which the results may be applicable to them are debatable. Furthermore, although the response rate was within a reasonable range for this type of study, regarding moral distress and the situations associated with it, we know nothing of the thoughts and experiences of nurses who did not take part. And it must be noted that interpretation of these results may be limited by social desirability bias regarding professional ethics, with a corresponding distortion of the results (Holmes, 2009).

## 5 CONCLUSIONS

The results reported here form a compelling argument that moral distress should be incorporated into the monitoring of nursing‐relevant context factors in hospital work settings. The chosen mixed methods design benefitted us considerably in developing our questionnaire on moral distress and provided a theoretical foundation on which we calculate with the help of the Rasch analysis an overall score in the form of an interval scale. In future studies relating to ongoing monitoring of nursing‐relevant factors of the hospital work setting, this will increase the MDS's usefulness. Finally, by supporting nurse managers to develop appropriate interventions to reduce the incidence, severity and consequences of moral distress, its results will help improve the quality of the work environment and nursing care.

## ACKNOWLEDGEMENT

We thank all the nurses and nurse managers at the hospitals that kindly agreed to participate in this study. We also thank all the funding institutions for their important financial support. MK is a PhD Student at the Department of Nursing Science, Faculty for Health, University of Witten/Herdecke in Witten, Germany. I would like to express my gratitude to my supervisors for their advice and their ongoing very helpful support.

## CONFLICT OF INTEREST

The authors report no conflict of interests.
